# Oxytocin and vasopressin flatten dominance hierarchy and enhance behavioral synchrony in part via anterior cingulate cortex

**DOI:** 10.1038/s41598-018-25607-1

**Published:** 2018-05-29

**Authors:** Yaoguang Jiang, Michael L. Platt

**Affiliations:** 1 Department of Neuroscience, Perelman School of Medicine, University of Pennsylvania, Philadelphia, PA USA; 2 Department of Psychology, School of Arts and Sciences, University of Pennsylvania, Philadelphia, PA USA; 3 Marketing Department, The Wharton School, University of Pennsylvania, Philadelphia, PA USA

## Abstract

The neuropeptides oxytocin (OT) and arginine vasopressin (AVP) influence social functions in many mammals. In humans and rhesus macaques, OT delivered intranasally can promote prosocial behavior in certain contexts. Yet the precise neural mechanisms mediating these behavioral effects remain unclear. Here we show that treating a group of male macaque monkeys intranasally with aerosolized OT relaxes their spontaneous social interactions with other monkeys. OT reduces differences in social behavior between dominant and subordinate monkeys, thereby flattening the status hierarchy. OT also increases behavioral synchrony within a pair. Intranasal delivery of aerosolized AVP reproduces the effects of OT with greater efficacy. Remarkably, all behavioral effects are replicated when OT or AVP is injected focally into the anterior cingulate gyrus (ACCg), a brain area linked to empathy and other-regarding behavior. ACCg lacks OT receptors but is rich in AVP receptors, suggesting exogenous OT may shape social behavior, in part, via nonspecific binding. Notably, OT and AVP alter behaviors of both the treated monkey and his untreated partner, consistent with enhanced feedback through reciprocal social interactions. These findings bear important implications for use of OT in both basic research and as a therapy for social impairments in neurodevelopmental disorders.

## Introduction

Oxytocin (OT) is an evolutionarily conserved neuropeptide that has been implicated in many important mammalian behaviors including maternal care^1–4^ and pair-bonding^5–10^. In recent years, there has been rapidly growing interest in the role of OT in human social behavior^11–18^. A single intranasal dose of OT in healthy humans enhances trust^19^, generosity^20^, and empathy^21^. In addition, OT facilitates social interaction by increasing the time people spend looking at each other’s eyes^22,23^, promoting attention to emotional cues^24^, and improving the ability to infer the mental states of others^25^ and identify their emotions^26,27^. OT also partially rescues social impairments in people with neuropsychiatric conditions such as autism, fragile X syndrome, schizophrenia, and social anxiety^28–33^.

For these reasons, OT is commonly interpreted, in both the neuroscientific literature and popular culture, as the ‘prosocial’ neuropeptide. A closer examination of the available evidence, however, suggests the effects of OT depend strongly on behavioral context^34^. For example, although OT can increase trust^19^, this effect is blunted by interacting with a partner who is unfamiliar^35^ or portrayed as untrustworthy^36^. Furthermore, OT can increase negative social judgments^37^, heighten out-group bias^38^, and amplify anxiety to unpredictable threat^39^. Finally, the effects of OT can vary as a function of gender, personality, attachment styles, and psychopathology^40–42^.

Our incomplete understanding of the role of OT in human social behavior partially results from our limited knowledge of its functional neurobiology. Human imaging studies suggest that intranasally delivered OT modulates BOLD signals in the amygdala as well as various regions in frontal cortex, midbrain, and striatum^11,12,43^, all of which have been linked to social behavior^44–46^. Evidence suggests the amygdala directly mediates OT influences on social behavior in rodents^47,48^ and non-human primates^49^, but the functional contributions of other regions, especially cortical areas implicated in social behavior—the so-called “social brain network”—remain unknown.

Like OT, arginine vasopressin (AVP) also shapes mammalian social behavior^50,51^. In rodents, AVP is known to contribute to a wide range of behaviors from aggression to affiliative behaviors such as pair-bonding and parental care^5,50–52^. In contrast, the effects of AVP on primate behavior are not completely clear. Several studies have reported that inhaling AVP can influence social behavior and modulate BOLD signal in humans^12,53,54^. AVP has also been linked to various psychiatric disorders such as anxiety, depression, and autism^29,55,56^. By comparison with the wealth of studies on OT effects in humans, however, there are very few studies systematically investigating the behavioral and physiological effects of AVP.

The functional neurobiology of OT and AVP in human social behavior is thus remarkably uncertain. This gap in our knowledge poses several serious problems. First, OT and AVP (as well as their receptor agonists or antagonists) are widely embraced as potential therapies for social impairments in disorders like autism, and dozens of clinical trials are underway in the US for precisely this purpose (refer to https://clinicaltrials.gov for a complete list of ongoing clinical trials, including OT trials at Massachusetts General Hospital, Emory University, an AVP trial at Stanford University, and an AVP antagonist trial at Hoffmann-La Roche) (also see^32,51,57^ for recent progress regarding translational research on OT and AVP). Yet the impact of these nonapeptides on real-world social behavior remains unclear. Furthermore, due to strong similarity in molecular structure, OT can bind to AVP receptors with high affinity and vice versa^58–60^, suggesting potential interactions between the two systems when either nonapeptide is delivered at high concentration. Yet the behavioral effects of OT and AVP are never directly compared in the same subjects in the same behavioral context.

To address these limitations in our understanding of OT and AVP neurobiology, we examined the effects of inhaling aerosolized OT and AVP on spontaneous social behavior in male rhesus macaques (see Jiang &Platt, forthcoming, for a comparable study in female rhesus macaques). Like humans, macaques live in large, hierarchical, mixed-sex groups^61^, engage in complex social interactions involving cooperation, empathy, and strategic behavior^62,63^, and use visual displays to communicate and guide such interactions^64^. In this study we found that intranasal treatment with OT relaxed social interactions between monkeys, thereby flattening the social hierarchy. OT also enhanced the temporal synchrony of reciprocal behaviors between monkeys, perhaps through increased attention or improved communication. Intranasal AVP reproduced all of these effects but with greater efficacy.

In addition to resembling humans in their social behaviors, macaque monkeys also exhibit a complex network of cortical and subcortical brain areas that appear to be homologous with the social brain network in humans^65–68^. Previous research in our lab showed that injecting OT into amygdala enhances social attention and promotes prosocial decisions in rhesus macaques, but injections into dorsolateral prefrontal cortex do not^49^. Here we extended these findings by injecting OT or AVP focally into the anterior cingulate gyrus (ACCg), a brain area implicated in empathy, social learning^68–71^, and computations of ‘other-oriented’ information^72–75^. Despite the small amounts delivered, injections of both OT and AVP into ACCg reproduced most of the effects of intranasal delivery. Because ACCg lacks OT receptors^76^, these findings indicate that OT may shape social behavior in part via nonspecific binding to AVP receptors. Overall, our findings strongly indicate that the effects of OT on social interactions in primates are mediated, in part, by modulating the neural circuit responsible for social deliberation and other-regarding consideration.

## Results

### Intranasal oxytocin (OT) relaxes social interaction

**Figure 1:**
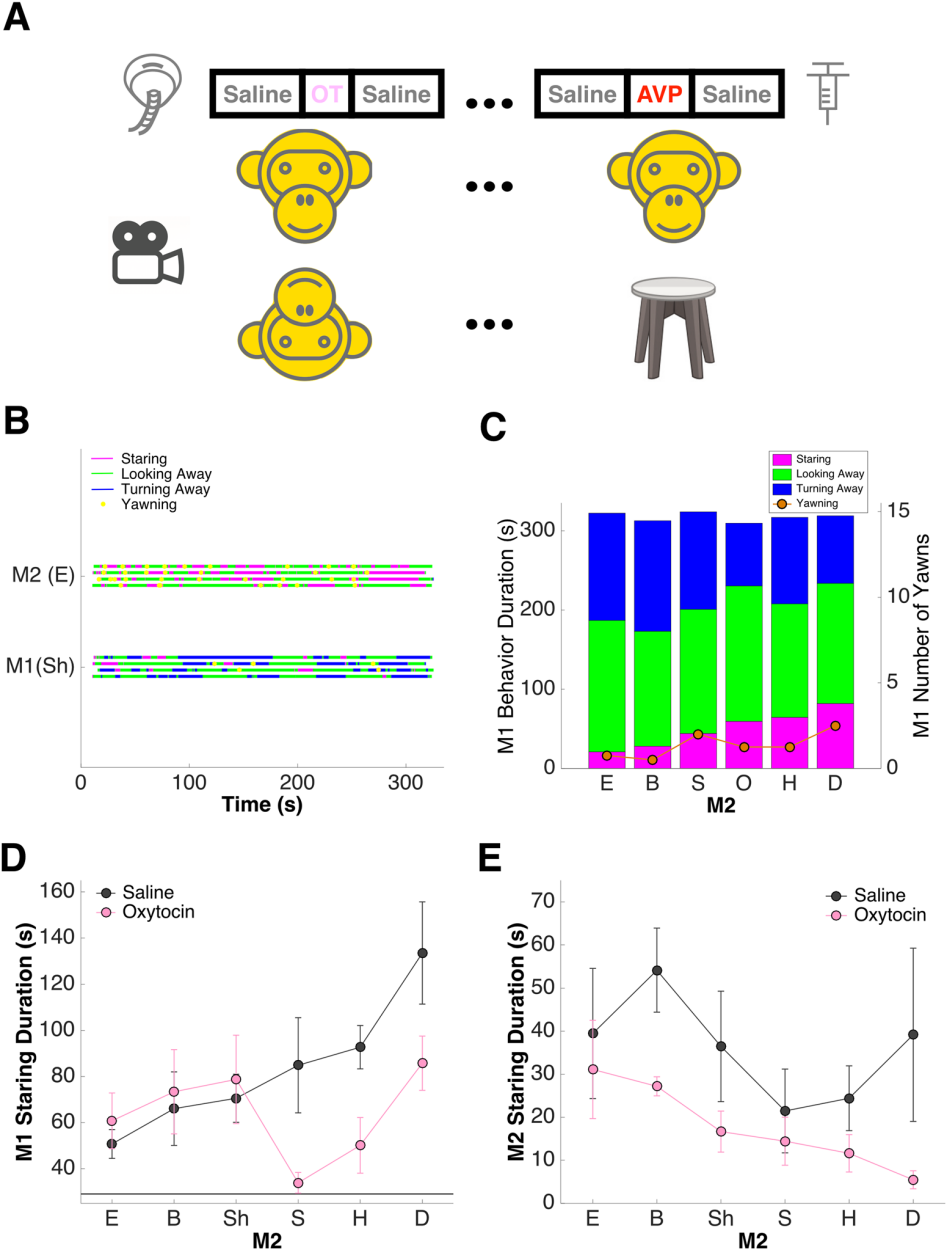
Ethograms, quantifiable behaviors, and effects of oxytocin (OT) inhalation. (**A**) Experimental design: one monkey (M1) receive saline, OT, or arginine-vasopressin (AVP) treatments via intranasal nebulization or intracortical injection prior to facing another monkey (M2) or an empty chair for 5 minutes in close proximity. (**B**) Example set of ethograms from one monkey pair facing each other in 4 saline sessions. M1 (Sh) inhaled saline; M2 (E) did not. (**C**) Summary of behaviors demonstrated by the same M1 (Sh) facing different M2s under saline. X axis: M2 identities, ordered by the time M1 spent staring at each. Y axis (left): average M1 behavior (i.e. staring, looking away, turning away) durations. Y axis (right): average M1 behavior (i.e. yawning) frequency. (**D**) For the same M1 (O), compared with saline, OT inhalation reduces overall staring time by M1. Black horizontal line: average time M1 spent staring at an empty chair; error bars: mean ± SEM. (**E**) For the same M1 (O), OT inhalation also reduces overall staring time by his untreated partners (M2s). Error bars: mean ± SEM.

Most prior studies of nonapeptides in human and nonhuman primates have focused on examining task performance in the laboratory, thus limiting ecological validity and translational potential. For this reason, we decided to probe the effects of OT and AVP on spontaneous, naturally-occurring social behaviors (though still in a laboratory setting). We recorded a series of 5-minute long videos of pairs of adult male macaque monkeys facing each other in close proximity. They were free to interact without danger of physical contact (see Fig. 1A as well as Methods, as well as Supplementary video). Prior to each session, one monkey (M1) inhaled either saline or OT via a pediatric nebulizer^77^, whereas the other monkey (M2) did not receive any treatment. Both monkeys’ behaviors were rated offline by 1–3 independent observers and subsequently converted to a set of ethograms (overall concordance across observers = 0.86, see Methods).

Figure 1B depicts example ethograms from one monkey pair in 4 consecutive saline sessions. Within this pair, M1 (Sh) was subordinate to M2 (E), frequently turning away from him (a sign of submission; M1 = 135.35 ± 24.99 s; M2 = 0.23 ± 0.23 s; df = 6, P = 0.028, Wilcoxon rank sum test), while M2 frequently stared at M1 (a sign of dominance; M1 = 20.90 ± 5.66 s; M2 = 112.85 ± 12.16 s; df = 6, P = 0.029, Wilcoxon rank sum test) (see Supplementary Figure 1A for another set of example ethograms). When the same M1 (Sh) faced less dominant M2s (Fig. 1C, from left to right), the duration of his stare systematically increased (F(5) = 2.79, P = 0.049; 1-way ANOVA). Following OT inhalation, M1 spent less time staring at other monkeys (saline = 83.09 ± 8.41 s; OT = 63.78 ± 6.86 s; df = 46, P = 0.024, Wilcoxon signed rank test) (Fig. 1D), and in turn his untreated partners also spent less time staring at him (saline = 35.86 ± 7.07 s; OT = 17.74 ± 3.09 s; df = 46, P = 0.025, Wilcoxon signed rank test) (Fig. 1E).

**Figure 2:**
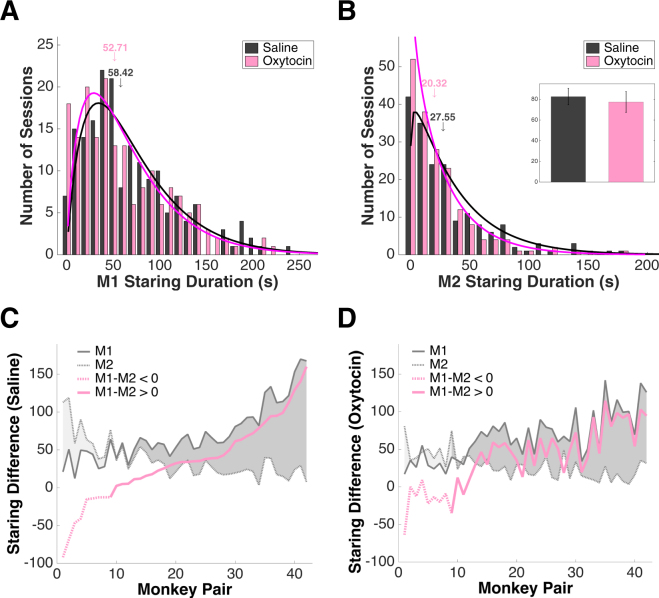
OT inhalation decreases staring and flattens social hierarchy. (**A)** Overall, OT treatment reduces staring by M1 (n = 180 face-off sessions * 2 treatment conditions). Black line: gamma fit of saline distribution; magenta line: gamma fit of OT distribution; arrows indicate medians. (**B)** OT treatment also reduces overall staring time by M2. Black line: gamma fit of saline distribution; magenta line: gamma fit of OT distribution; arrows indicate medians. Insert: Compared with saline (grey), OT (pink) does not change M1’s staring at an empty chair. Error bars: mean ± SEM. (**C**) As M1 staring increases, M2 staring decreases, and the difference between the two widens correspondingly. X axis: monkey pairs ordered according to the difference between M1 and M2 staring time; left to right corresponds to increasing dominance of M1 over M2. (**D**) OT inhalation narrows the gap between M1 and M2 staring durations and flattens the dominance hierarchy. X axis: monkey pairs in the same order as in (**C**).

For the population (n = 7 monkeys, 360 face-off sessions), the overall effect of OT was a small reduction in staring duration by the treated monkey (M1 staring, saline = 71.30 ± 3.69 s, median = 58.42 s; OT = 65.73 ± 3.67 s, median = 52.71 s; df = 358, P = 0.098, Wilcoxon rank sum test) (Fig. 2A). Remarkably, OT treatment of M1 also altered the behavior of his untreated partner, who showed a significant reduction in staring as well (M2 staring, saline = 36.84 ± 2.89 s, median = 27.55 s; OT = 28.11 ± 2.21 s, median = 20.32 s; df = 358, P = 0.031, Wilcoxon rank sum test) (Fig. 2B). OT specifically reduced the average time per staring bout, for both M1 (saline = 6.42 ± 0.45 s; OT = 5.82 ± 0.54 s; df = 5758, P = 0.095, Wilcoxon rank sum test) and M2 (saline = 7.62 ± 0.62 s; OT = 6.39 ± 0.58 s; df = 3130, P = 0.048, Wilcoxon rank sum test), but did not alter the number of staring bouts per session (M1 number of stares, saline = 15.90 ± 0.66; OT = 16.19 ± 0.71; df = 358, P = 0.775, Wilcoxon rank sum test; M2 number of stares, saline = 9.33 ± 0.55; OT = 8.54 ± 0.57; df = 358, P = 0.148, Wilcoxon rank sum test). Importantly, the average time M1 spent staring at an empty chair was the same under saline and OT conditions, indicating OT has a specifically social effect, as opposed to merely altering visual scanning behavior in general (saline = 82.62 ± 7.90 s; OT = 78.28 ± 10.45 s; df = 59, P = 0.741, Wilcoxon rank sum test) (Fig. 2B, insert).

Across all possible monkey pairs (n = 7*6 = 42 pairs), M1 staring increased with dominance, M2 staring decreased correspondingly, and the difference between the two widened, reflecting the existing social hierarchy (Fig. 2C). OT narrowed this gap and diminished the difference in staring durations, suggesting a flatter dominance hierarchy (M1-M2 staring difference, saline = −91.96 to 160.00 s, interquartile range = 63.49 s, slope = 4.21; OT = −63.66 to 114.53 s, interquartile range = 54.84 s, slope = 3.33) (Fig. 2D). OT significantly reduced staring by highly dominant M1s as well as untreated highly dominant M2s (Supplementary Figure 2A-B). OT also evoked a non-significant reduction in turning away duration by the treated monkey (M1 turning away under saline = 34.76 ± 3.41 s; under OT = 30.79 ± 3.19 s; df = 358, P = 0.108, Wilcoxon rank sum test), but not his untreated partner (M2 turning away under saline = 68.46 ± 5.17 s; under OT = 72.17 ± 5.40; df = 358; P = 0.708, Wilcoxon rank sum test). OT did not change the frequency of other, more aggressive behaviors such as threats (Supplementary Figure 2C).

### OT enhances behavioral synchrony

**Figure 3:**
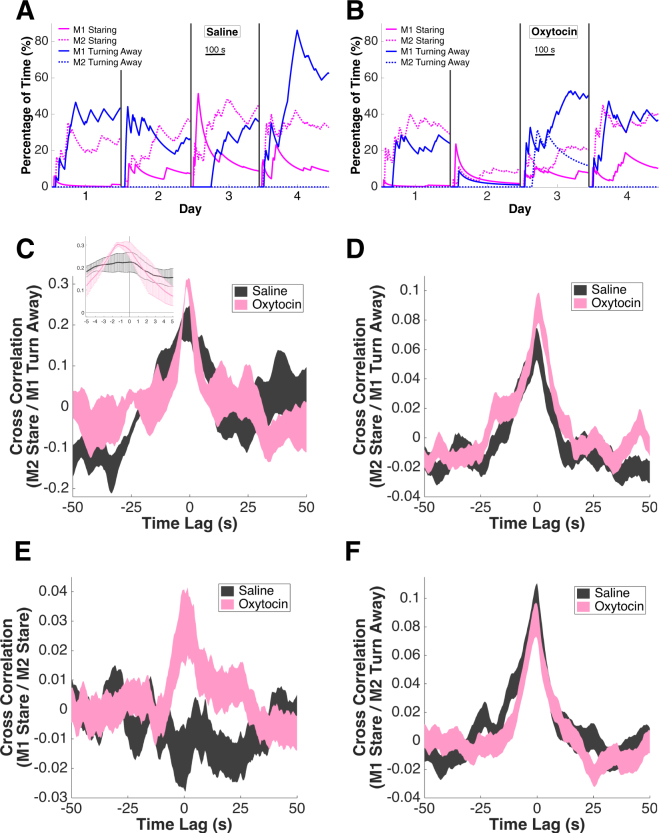
OT inhalation increases behavioral synchrony. (**A)** Example set of temporal dynamics for behaviors from one monkey pair (M1: Sh; M2: E) in 4 saline sessions (the same as Fig. 1A). Black vertical lines mark individual sessions. (**B**) Example set of temporal dynamics for behaviors from the same pair of monkeys in 4 OT sessions. Black vertical lines mark individual sessions. (**C**) For the same monkey pair (M1: Sh; M2: E), OT treatment increases the cross correlation between M2 staring and M1 turning away. Thickness of the curves indicates mean ± SEM. Insert: The same cross correlation under saline (grey) and OT (pink) plotted on a finer time scale ( ± 5 s). Error bars: mean ± SEM. (**D**) For the population, OT treatment increases the cross correlation between M2 staring and M1 turning away. Thickness of the curves indicates mean ± SEM. (**E**) OT also increases the cross correlation between M1 and M2 staring. Thickness of the curves indicates mean ± SEM. (**F**) OT does not, however, change the cross correlation between M1 staring and M2 turning away. Thickness of the curves indicates mean ± SEM.

Recent social neuroscientific studies have linked behavioral and neural synchrony to stronger social bonds^78–81^, and nonapeptide signaling may contribute to such social synchrony^82–84^. To address this possibility, we examined the temporal correlation of behaviors within pairs of monkeys following saline or OT delivery. Figure 3A shows an example set of behavioral dynamics from two monkeys (M1: Sh, subordinate; M2: E, dominant) in 4 saline sessions (as in Fig. 1A); Fig. 3B depicts the same pair in 4 OT sessions. OT increased the temporal coupling of subordinate M1’s turning away behavior with dominant M2’s staring (cross correlation at time −1 s, saline = 0.20 ± 0.04; OT = 0.30 ± 0.01; df = 6, P = 0.150, Wilcoxon rank sum test) (Fig. 3C and insert). Across all pairs, OT consistently boosted the temporal coupling between M1 turning away and M2 staring (cross correlation at time 0, saline = 0.06 ± 0.01; OT = 0.09 ± 0.01; df = 358, P = 0.089, Wilcoxon rank sum test) (Fig. 3D, see Supplementary Figure 2D for zoomed in version). After OT, M1 staring and M2 staring also became more tightly coupled in time, indicating that under OT M1 was more likely to return M2’s stare (cross correlation at time 0, saline = −0.02 ± 0.01, OT = 0.03 ± 0.01; df = 358, P = 0.011, Wilcoxon rank sum test) (Fig. 3E, see Supplementary Figure 2E for zoomed in version). By contrast, the cross correlation between M1 staring and M2 turning away was not affected by OT, as M2 did not receive any OT treatment (cross correlation at time 0, saline = 0.10 ± 0.01, OT = 0.08 ± 0.01; df = 358, P = 0.383, Wilcoxon rank sum test) (Fig. 3F, see Supplementary Figure 2F for zoomed in version). The autocorrelations of M1’s staring and turning away were not influenced by OT (M1 staring at time ± 1 s, saline = 0.65 ± 0.02; OT = 0.61 ± 0.02; df = 59, P = 0.214, Wilcoxon rank sum test; M1 turning away at time ± 1 s, saline = 0.74 ± 0.04; OT = 0.75 ± 0.05; df = 59, P = 0.416, Wilcoxon rank sum test; empty chair sessions). Together, these findings demonstrate that the influence of OT on a treated monkey evoke behavioral changes in an untreated monkey. More specifically, under OT treatment, when faced with M2’s stare, M1 either immediately stares back (to show dominance) or quickly turns away (to show subordination), which in turn leads to reduced stare by M2. Thus under OT both monkeys become more responsive to each other and more efficient in communicating dominance status, perhaps through an increase in attention to social cues^72^.

### Intranasal arginine vasopressin (AVP) reproduced the effects of OT with greater efficacy

**Figure 4:**
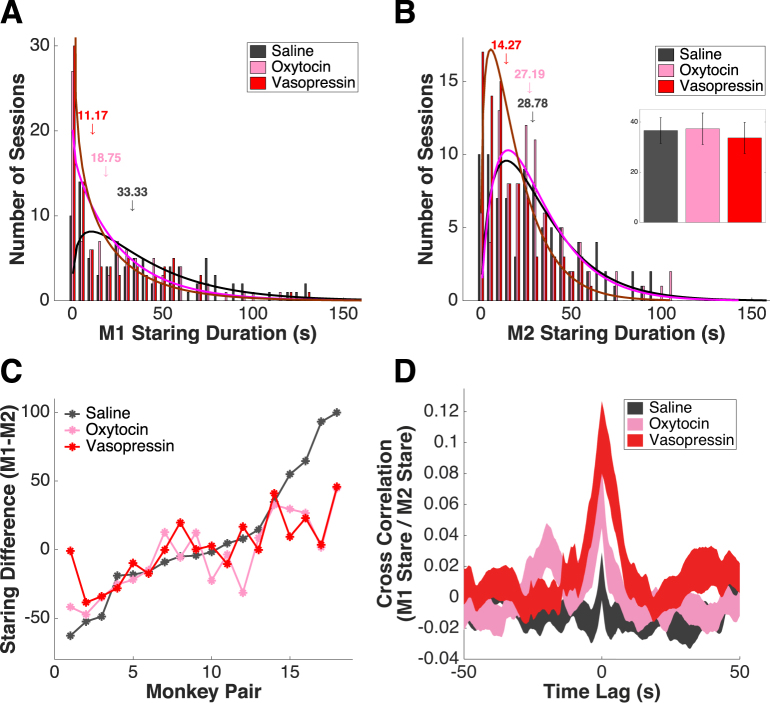
AVP inhalation also decreases staring, flattens social hierarchy, and enhances behavioral synchronization. (**A**) OT and AVP inhalations similarly reduce staring by M1 (n = 90 face-off sessions * 3 treatment conditions). Black line: gamma fit of saline distribution; magenta line: gamma fit of OT distribution; maroon line: gamma fit of AVP distribution; arrows indicate medians. (**B**) AVP but not OT inhalation significantly reduces overall staring by M2. Black line: gamma fit of saline distribution; magenta line: gamma fit of OT distribution; maroon line: gamma fit of AVP distribution; arrows indicate medians. Insert: Compared with saline (grey), neither OT (pink) nor AVP (red) alters M1 staring at an empty chair. Error bars: mean ± SEM. (**C**) Both OT and AVP inhalations reduce the difference between M1 and M2 staring. X axis: monkey pairs ordered by the difference in staring between M1 and M2 under saline; left to right corresponds to increasing dominance of M1 over M2. (**D**) OT and AVP both enhance the cross correlation between M1 and M2 staring. Thickness of the curves indicates mean ± SEM.

In a new colony of 7 monkeys (M1 = 3 males, M2 = 3 males, 4 females), we systematically compared the effects of OT and AVP inhalation at the same concentration (25 IU delivered in 1 ml saline vehicle). We reconfirmed in this group that OT inhalation significantly reduced staring by the treated monkey, and found that AVP inhalation had a similar effect (saline = 42.48 ± 4.21 s, median = 33.33 s; OT = 26.86 ± 2.88 s, median = 18.75 s; AVP = 22.15 ± 2.74 s, median = 11.17 s; 1-way ANOVA, F(2,267) = 10.15, P = 0.000; multiple comparison, saline vs OT, P = 0.003; saline vs AVP, P = 0.000) (Fig. 4A). Although OT treatment of M1 also resulted in an insignificant reduction in untreated M2’s staring, AVP treatment of M1 significantly reduced staring by M2 (saline = 34.85 ± 3.21 s, median = 28.78 s; OT = 31.33 ± 2.39 s, median = 27.19 s; AVP = 19.23 ± 1.72 s, median = 14.27 s; 1-way ANOVA, F(2,267) = 10.63, P = 0.000; multiple comparison, saline vs OT, P = 0.14; saline vs AVP, P = 0.004) (Fig. 4B). Overall, AVP was more effective than OT in reducing staring by M1 and M2 (1-way ANOVA, F(2,537) = 18.46, P = 0.000; multiple comparison, saline vs OT, P = 0.004; saline vs AVP, P = 0.000; OT vs AVP, P = 0.013). In neither condition did treatment change M1 staring at an empty chair (saline = 36.60 ± 5.06 s; OT = 37.31 ± 6.25 s; AVP = 33.64 ± 6.16 s; 1-way ANOVA, F(2,42) = 0.11, P = 0.896) (Fig. 4B, insert). Across all monkey pairs (n = 3*6 = 18 pairs), both OT and AVP flattened the pre-existing social hierarchy (M1-M2 staring difference, saline = −62.68 to 99.76 s, interquartile range = 53.03 s, slope = 8.29; OT = −47.10 to 44.61 s, interquartile range = 38.00 s, slope = 5.09; AVP = −38.52 to 70.99 s, interquartile range = 27.07 s, slope = 4.68) (Fig. 4C). Both OT and AVP significantly reduced staring by dominant M1s and M2s, but effects of AVP were stronger (Supplementary Figure 3A-B). Finally, after treatment with either OT or AVP, M1 staring and M2 staring were more tightly coupled in time (cross correlation at time 0, saline = 0.01 ± 0.02; OT = 0.07 ± 0.02; AVP = 0.10 ± 0.02; 1-way ANOVA, F(2,229) = 4.45, P = 0.013) (Fig. 4D, see Supplementary Figure 3C for zoomed in version). The autocorrelation of M1 staring was unaffected by either OT or AVP (at time ± 1 s, saline = 0.83 ± 0.03; OT = 0.85 ± 0.03; AVP = 0.83 ± 0.03; F(2,42) = 0.22; P = 0.801, 1-way ANOVA; empty chair sessions) (Supplementary Figure 3D).

### The effects of OT and AVP are partially mediated via anterior cingulate gyrus

**Figure 5:**
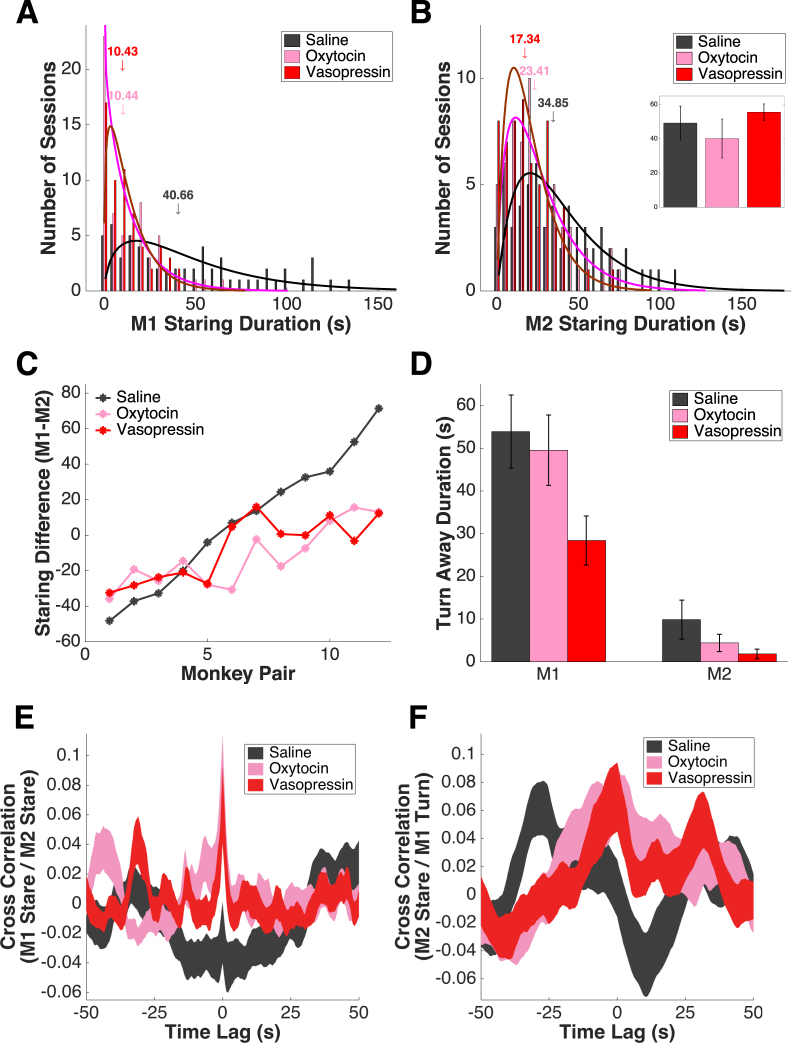
Focal injections of OT and AVP into ACCg reduce staring, flatten social hierarchy, and enhance behavioral synchronization. (**A**) Both OT and AVP injections reduce staring by M1 (n = 60 face-off sessions * 3 treatment conditions). Black line: gamma fit of saline distribution; magenta line: gamma fit of OT distribution; maroon line: gamma fit of AVP distribution; arrows indicate medians. (**B**) OT and AVP injections also reduce staring by M2. Black line: gamma fit of saline distribution; magenta line: gamma fit of OT distribution; maroon line: gamma fit of AVP distribution; arrows indicate medians. Insert: Neither OT (pink) nor AVP (red) treatment significantly change the average time (in s) M1 spent staring at an empty chair. Error bars: mean ± SEM. (**C**) Both OT and AVP injections in ACCg decrease the difference between M1 and M2 staring. X axis: monkey pairs ordered by staring difference between M1 and M2 under saline; left to right corresponds to increasing dominance of M1 over M2. (**D**) AVP but not OT injection also significantly reduces the turning away time of M1. Error bars: mean ± SEM. (**E**) OT and AVP injections into ACCg both enhance the cross correlation between M1 and M2’s staring. Thickness of the curves indicates mean ± SEM. (**F**) OT and AVP injections into ACCg also enhance the cross correlation between M2 staring and M1 turning away. Thickness of the curves indicates mean ± SEM.

We next investigated the neural circuitry mediating the effects of OT and AVP on social behavior. To do so, we injected OT or AVP (1 IU in 2 µl saline vehicle) focally into the anterior cingulate gyrus (ACCg), a distinct region within ACC implicated in social behavior^64–71^ (M1 = 2 males, M2 = 3 males, 4 females). We found both OT and AVP injections significantly reduced staring by the treated monkey (saline = 47.85 ± 4.92 s, median = 40.66 s; OT = 14.61 ± 1.95 s; median = 10.44 s; AVP = 13.78 ± 1.55 s, median = 10.43 s; 1-way ANOVA, F(2,267) = 37.3, P = 0.000; multiple comparison, saline vs OT, P = 0.000; saline vs AVP, P = 0.000) (Fig. 5A). Furthermore, both OT and AVP injections in M1 reduced staring by M2 (saline = 39.93 ± 3.61 s, median = 34.85 s; OT = 26.65 ± 2.43 s, median = 23.41 s; AVP = 20.97 ± 1.93 s, median = 17.34 s; 1-way ANOVA, F(2,267) = 12.54, P = 0.000; multiple comparison, saline vs OT, P = 0.002; saline vs AVP, P = 0.000) (Fig. 5B) (See Supplementary Figure 4A-B for an additional set of OT injection data in the first monkey colony). Neither treatment changed M1 staring at an empty chair (saline = 49.19 ± 9.83 s; OT = 40.13 ± 11.39 s; AVP = 55.55 ± 4.81 s; 1-way ANOVA, F(2,27) = 0.72, P = 0.495) (Fig. 5B, insert). Across all monkey pairs (n = 2*6 = 12 pairs), both OT and AVP injections flattened the social hierarchy (M1-M2 fixation difference, saline = −48.20 to 71.31 s, interquartile range = 60.55 s, slope = 10.34; OT = −35.85 to 15.57 s, interquartile range = 29.68 s, slope = 4.78; AVP = −32.31 to 21.08 s, interquartile range = 33.30 s, slope = 5.00) (Fig. 5C). Both OT and AVP injections significantly reduced staring by dominant M1s and M2s (Supplementary Figure 4C-D).

Additionally, AVP but not OT injection significantly reduced turning away by M1 but not by M2 (M1 saline = 53.89 ± 8.58 s; OT = 49.55 ± 8.24 s; AVP = 28.39 ± 5.74 s; 1-way ANOVA, F(2,177) = 3.2, P = 0.043; multiple comparison, saline vs OT, P = 0.914; saline vs AVP, P = 0.047; M2 saline = 9.86 ± 4.57 s; OT = 4.41 ± 2.03 s; AVP = 1.84 ± 1.13 s; 1-way ANOVA, F(2,177) = 1.92, P = 0.150) (Fig. 5D). Finally, both OT and AVP injections boosted the temporal coupling of staring by M1 and M2 (cross correlation at time 0, saline = −0.02 ± 0.02; OT = 0.09 ± 0.03; AVP = 0.07 ± 0.03; 1-way ANOVA, F(2,158) = 4.89, P = 0.009) (Fig. 5E, see Supplementary Figure 4E for zoomed in version). OT and AVP also boosted the correlation between M1 turning away and M2 staring (cross correlation at time 0, saline = 0.00 ± 0.02; OT = 0.07 ± 0.02; AVP = 0.07 ± 0.02; 1-way ANOVA, F(2,77) = 2.74, P = 0.071) (Fig. 5F, see Supplementary Figure 4F for zoomed in version). By contrast, the autocorrelations of M1’s staring and turning away were not influenced by either treatment (M1 staring at time ± 1 s: under saline = 0.85 ± 0.02, under OT = 0.77 ± 0.05; under AVP = 0.80 ± 0.02; F(2,26) = 1.21; P = 0.220, 1-way ANOVA; M1 turning away at time ± 1 s: under saline = 0.94 ± 0.02 under OT = 0.96 ± 0.00; under AVP = 0.98 ± 0.00; F(2,9) = 0.77; P = 0.490, 1-way ANOVA; empty chair sessions).

## Discussion

Oxytocin shapes social behavior in a variety of animals, from rodents like mice^85–87^ and prairie voles^5,7,8^, to nonhuman primates like marmosets^9^, squirrel monkeys^88^, titi monkeys^89^, rhesus macaques^49,77,90,91^, and chimpanzees^92^. The overwhelming evidence supporting a functional role for OT in animal social behavior has greatly motivated the surge of interest in OT function in humans^87,93^. The effects of acute OT administration in humans, however, remain highly controversial. Though early studies reported OT enhances prosocial behavior and improves social cognition, recent meta-analyses suggest that almost half of studies failed to find a significant effect of OT on human social behavior^13–16^. By contrast, using a dose (25 IU) comparable to those used in most human studies^11–14^, we found OT consistently and robustly relaxed social interactions, flattened the social hierarchy, and enhanced social communication in rhesus macaque monkeys.

Our results add to the long line of positive findings in the effectiveness of OT in rodents and nonhuman primates, but they also raise questions regarding the potential source of variability in OT research in humans. Firstly, OT often has task or stimulus specific effects in humans^94–98^, making it difficult to compare across studies^99,100^. OT also interacts with individual traits such as gender, personality, attachment styles, and psychopathology^34,40–42,101^. Here we focused solely on spontaneous interactions of adult male macaque monkeys living within a stable hierarchical social group, which provided us with a level of control that is rarely seen in human social studies, and may have contributed to the detectability of the effects of OT, as well as AVP.

Another source of variability in human studies is the method of delivery. In laboratory or clinical setting OT is typically applied via a nasal spray^102,103^. Intranasal OT spray in humans increases OT levels in the cerebrospinal fluid (CSF^104^) and, similarly, intranasal spray of AVP increases AVP in CSF^105^. In primates, however, compared with nasal spray, aerosolized OT delivered via nebulizer (as in our study) either more consistently^106^ or more effectively^107^ increases OT in CSF. Moreover, most studies in humans rely on participants to self-administer OT spray^102,103,108,109^. Lack of standardization in self-administration may have led to variation in the effectiveness of OT delivery in humans. By contrast, aerosolized delivery through nebulizer eliminates the need for user involvement, thus ensuring more consistent and thorough delivery. This may be critical for potential therapeutic use of OT in young children or psychiatric patients who have difficulty following instructions.

One other source of potential variability is the amount of OT used. Most human studies delivered 20–40 IUs of OT regardless of the subjects’ age, gender, or body weight^11–14^. Our study as well as previous studies of OT effects in nonhuman primates employed similar doses (25 IU in current study; 25 IU in 77, 91; 48 IU in 106; 24 IU in 107; 80 IU in^110^). The average weight of a rhesus macaque monkey, however, is much lower than that of a human subject, leading to the possibility of a higher concentration of CSF OT in macaques when the same amount of exogenous OT is delivered. To facilitate the comparison of OT effects across future studies and especially across species, OT dosage may need to be calculated on an IU per kilogram base^111^.

Comparison of our behavioral and injection results lends support to the hypothesis that inhaled OT influences social behavior at least partially through nonspecific binding with AVP receptors in medial frontal cortex. Because of similarity in molecular structure, OT can bind to AVP receptors with high affinity^58–60^. Indeed, in rodents OT and AVP can act on the ‘opposite’ receptor to influence behavior^112–114^. In nonhuman primates, the distribution of post-synaptic OT receptors (OXTR) is extremely sparse in frontal cortex^76^. By contrast, AVP receptors (V1a) are much more abundant in cortical areas implicated in social behavior, including prefrontal, cingulate, and entorhinal cortex, as well as in amygdala, hypothalamus, and brainstem^115,116^. Our study offers one of the first pieces of evidence strongly implicating the AVP system in what was traditionally considered to be oxytocinergic effects on primate social behavior. These results highlight the need to further investigate the interaction between AVP and OT systems through selective agonist or antagonist administration. For example, if targeted ACCg injections of AVP antagonist could partially abolish the behavioral effects of intranasally delivered OT, we will be able to rule out the possibility that local injections of OT impact behavior via different pathways than OT inhalation, and conclude with greater confidence that inhaled OT affects behavior through cortical AVP receptors. More generally, our results also emphasize the importance of further examination on the AVP system independent of OT, as we observed in many aspects AVP is more effective than OT in positively modulating social behaviors. Indeed, an ongoing clinical trial (Hoffmann-La Roche) has demonstrated the possibility of tackling the AVP system alone to rescue social deficients in autism disorder.

In our study, focal injections of both OT and AVP into ACCg recapitulated most of the behavioral effects of intranasal delivery. ACC has long been linked to social behavior in mammals^117–119^. ACCg is a specialized sub-region within ACC that is interconnected with various other brain areas in the ‘social brain network’, including the temporoparietal junction (TPJ), dorsolateral prefrontal cortex (dmPFC), ventromedial prefrontal cortex (vmPFC), and amygdala^120–123^. Resting-state connectivity of ACCg with these areas increases with the size of an individual’s social network^124,125^, and abnormal cytoarchitecture and functional connectivity of ACCg are prevalent in autism spectrum disorder (ASD^126,127^). Both single-unit recordings and neuroimaging studies suggest ACCg processes ‘other-oriented” information including the decisions made by others and the rewards and punishments they receive^68–75^. Our findings further endorse the idea that ACCg plays a fundamental role in natural social interactions^128^.

Finally, we discovered that altering the behavior of a monkey with either OT or AVP has a contagious effect on his untreated social partners, and thus the social environment in general. This significant modulation of others’ behavior is mediated through not only changes in behavioral frequency or duration, but also improved temporal synchrony across subjects. Thus, the prosocial effects of OT and AVP can be interpreted as an improvement in the efficiency of bidirectional social communication. These results directly support previous reports of OT improving behavioral synchrony^83,84^ and mutual gaze^129–131^ in neurotypical individuals as well as ASD patients, and thus bear important implications for use of social peptides in both basic research and as a therapy for social impairments in neurodevelopmental disorders.

## Methods

### Animals

Data reported in the first part of the Results (Figs 1–3, Supplementary Figures 1-2, 4A-B) was collected at Duke University. All procedures were correspondingly approved by the Institutional Animal Care and Use Committee of Duke University, and performed in accordance with their relevant guidelines and regulations. Seven male rhesus macaques (B, D, E, H, O, Sh, S) participated in the OT inhalation experiment at Duke. Each of them had equal probabilities of being the treated monkey (i.e. M1) or the untreated partner (i.e. M2). Seven male rhesus macaques (B, C, D, E, H, O, S) participated in the OT injection experiment. Among these seven, three (C, O, S) received OT injections in ACCg (i.e. M1), whereas the other four only participated as the untreated partner (i.e. M2). Monkey Sh participated in the entirety of the inhalation experiment but was replaced with monkey C for the injection experiment. The subjects lived in a colony of 12 male rhesus macaques. Cages were arranged facing toward the center of the room, along two walls, permitting all animals to be in continuous visual and auditory contact. All animals were between the ages of 6 and 15 at the time of the experiments and had been in the colony for at least a year.

The rest of the data reported in this paper was collected at University of Pennsylvania (Figs 4–5, Supplementary Figure 3,4C–F). All procedures were correspondingly approved by the Institutional Animal Care and Use Committee of the University of Pennsylvania, and performed in accordance with their relevant guidelines and regulations. Three male rhesus macaques (D, O, S) and four female rhesus macaques (B, C, F, Sch) participated in the OT and AVP inhalation experiment. All of them had equal probabilities of being M2, but only the three males participated as M1 and received treatments. The same seven monkeys also participated in the OT and AVP injection experiment, but only two male monkeys (D, S) participated as M1 and received injections in ACCg. These macaques lived in a colony together (with no other monkeys) for the duration of this experiment. Cages were arranged facing toward the center of the room, along two walls, permitting all animals to be in continuous visual and auditory contact. All animals were between the ages of 11 and 18 at the time of the experiments and had been in the colony for at least six months.

### Experimental Setup

In each experiment session, two monkeys faced each other face-on in an empty room, and were free to interact for 5 minutes. The monkeys sat in their respective primate chairs (Crist Instruments), and the two chairs were positioned close together without touching each other (~30 cm apart from edge to edge). A video camera (Logitech, 60 fps) was positioned on the right side of M1/ the left side of M2, and simultaneously recorded both monkeys’ behaviors into an MP4 file. On each day, one M1 faced six different M2s sequentially. In addition, the same M1 also faced an empty chair for 5 minutes. The order in which M1 faced the other monkeys together with the empty chair was determined randomly each day.

### Pharmacological Manipulation

The procedure for intranasal OT delivery in macaque monkeys has been described in detail previously^72,86,87^. Briefly, monkeys were trained to accept a pediatric nebulizer mask (Pari Labs) over the nose and mouth. Through the nebulizer 1 ml of OT solution (25 IU/ml in saline; Agrilabs/Sigma Aldrich) or saline was delivered at a constant rate (0.2 ml/min) over a total of 5 minutes. Behavioral testing began 30 minutes after intranasal delivery and continued for 1-2 hours. This OT dosage and timing protocol was similar to that typically used in humans^11–14^ and other non-human primate studies^72,87,103,104^. We followed the same procedure for intranasal AVP delivery (25 IU in 1 ml saline; Sigma Aldrich). The same amount of neuropeptide (25 IU) was delivered to all monkeys regardless of their weights (ranging from 10–16 kg at the time of the experiment), which resulted in a dosage of ~1.5–2.5 IU/kg. Neuropeptide and saline treatments were delivered on alternating days, with each monkey receiving no more than 5 treatments per week. The same monkey never received OT and AVP treatment within the same week. The order of treatments was counterbalanced across monkeys as well as within monkeys between weeks to mitigate any possible order effects. Furthermore, to rule out the possibility that the particular sequence with which different monkey pairs were tested (after saline or neuropeptide delivery) had any impact on behavior, 1-way ANOVAs were performed on different behaviors. The testing order here was treated as a nominal variable with 6 levels (with 1 corresponding to the first pair tested and 6 corresponding to the last pair tested on the same day). This analysis did not reveal any significant order effect (for example, for staring duration under saline-OT-AVP conditions, 1-way ANOVA, F(5,264) = 1.03, P = 0.400). In addition, general linear models were constructed for different behaviors as well, with the testing order being treated as a continuous variable and measured as the estimated time passed from drug administration to behavioral testing for each pair. This analysis revealed no significant order effect either.

The procedure for OT injection has also been described previously^49^. For the three monkeys receiving OT injections, the location of their ACCg was first identified by a series of structural magnetic resonance images (MRI; 3 T, 1-mm slices) and then confirmed with a set of preliminary electrophysiological recordings. On each experiment day, 2 μL of OT solution (0.5 IU/μL of OT in saline; Agrilabs/Sigma Aldrich) or saline was injected into the ACCg via a Hamilton microsyringe (Hamilton). To minimize tissue damage, all injections were delivered at a rate of 0.2 μL/min and restricted unilaterally in each animal (C, O: right; D, S: left). The procedure for AVP injection (1 IU in 2 μL saline; Sigma Aldrich) was the same as OT injection. Neuropeptide and saline injections were delivered on alternating days, with each monkey receiving no more than 1 injection every other day. The same monkey never received OT and AVP injections within the same week. The order of treatments was counterbalanced across monkeys as well as within monkeys between weeks to mitigate any possible order effects (for example, for staring duration under saline-OT-AVP conditions,1-way ANOVA, F(5,174) = 1.68, P = 0.142). For the same monkey, inhalation and injection treatments were always delivered in distinct time periods (at least 2 weeks apart).

### Data Analysis

Each behavioral video was rated offline by 1 to 3 independent viewers, all of whom were blind to treatment conditions. Viewers used a Python GTK based, custom GUI to play and pause the video, adjust its speed, and code monkey behaviors by pressing a set of keys on the keyboard. The string of keyboard inputs and their corresponding time stamps were imported into MATLAB (Mathworks) and converted into a pair of behavioral ethograms via custom MATLAB scripts. When more than one viewer rated the same video (~50% of all the videos), their ratings were averaged to generate the ethograms. For the same videos rating consistency was very high across different viewers (for fixation duration: r = 0.58, df = 184, P = 0.000; for number of fixations: r = 0.24, df = 202, P = 0.001; for turning away duration: r = 0.65, df = 184, P = 0.000; for number of turning aways: r = 0.41, df = 184, P = 0.000; for number of yawns: r = 0.80, df = 184, P = 0.000; for number of threats: r = 0.91, df = 184, P = 0.000). The overall concordance across observers was 0.86 (0.88 between DX and YJ, 0.79 between DX and JF, 0.91 between YJ and EZ, 0.82 between YJ and SRM). All subsequent data analyses were accomplished with custom MATLAB scripts.

All statistical tests were two-tailed. For hypothesis testing between two samples, a non-parametric Wilcoxon signed rank test (for paired samples) or Wilcoxon rank sum test (for un-paired samples) was used. For comparison among more than two samples, an ANOVA was used together with multiple comparisons (Tukey’s HSD test) when appropriate. Correlation coefficients were estimated with Pearson’s r. All behavior histograms were fitted with Gamma distributions:

1 $$y=f(x|a,b)=\frac{1}{{b}^{a}{\rm{\Gamma }}(a)}{x}^{a-1}{e}^{\frac{-x}{b}}$$

where Γ is the Gamma function (${\rm{\Gamma }}(n)=(n-1)!$), a is a shape parameter, and b is a scale parameter. Cross correlations and auto-correlations were calculated in non-overlapping 100 ms windows.

### Data Availability

The datasets generated during and/or analyzed during the current study are available from the corresponding author upon request.

## Electronic supplementary material

Supplementary figures and legends

Supplementary video
