# Supplementary material for: Oxytocin and vasopressin flatten dominance hierarchy and enhance behavioral synchrony in part via anterior cingulate cortex
Source: Sci Rep. 2018 May 29;8:8201. doi: 10.1038/s41598-018-25607-1 (PMC5974023; doi:10.1038/s41598-018-25607-1)
Supplement: Supplementary file 1 — Supplementary figures and legends [file 41598_2018_25607_MOESM1_ESM.pdf]

Title

Oxytocin and vasopressin flatten dominance hierarchy and enhance behavioral synchrony in part via anterior cingulate cortex

Authors

Yaoguang Jiang, Michael L. Platt

Supplementary Figure Legends

Figure S1: Additional example ethograms and relationships between various quantifiable behaviors. **A:** Example sets of ethograms for the same monkey pair (M1: H; M2: E) facing each other in 4 saline sessions. **B:** Across the population, M1 staring and M1 looking away are negatively correlated (r = -0.41, df = 82, P = 0.000). **C:** M1 staring and turning away are also negatively correlated (r = -0.35, df = 82, P = 0.001). **D:** M1 staring and M2 staring are negatively correlated (r = -0.38, df = 82, P = 0.000).

Figure S2: Effects of OT inhalation on behavior. **A:** OT reduces staring by highly dominant M1s (low dominance M1, saline = 38.62 ± 3.49 s; OT = 40.04 ± 4.95 s; middle dominance M1, saline = 61.60 ± 1.39 s; OT = 64.88 ± 4.65 s; high dominance M1, saline = 114.73 ± 8.63 s; OT = 92.57 ± 8.87 s; df = 13, P = 0.030, Wilcoxon signed rank test on high dominance M1s). Error bars: mean ± SEM; dominance measured by average staring durations under saline. **B:** OT reduces staring by highly dominant M2s (low dominance M2, saline = 15.35 ± 1.45 s; OT = 18.54 ± 3.51 s; middle dominance M2, saline = 30.67 ± 1.50 s; OT = 25.43 ± 3.39 s; high dominance M2, saline = 63.83 ± 7.11 s; OT = 39.81 ± 5.14 s; df = 13, P = 0.000, Wilcoxon signed rank test on high dominance M2s). Error bars: mean ± SEM; dominance measured by average staring durations under saline. **C:** OT treatment does not change yawning or threats by M1, compared with saline (M1 number of yawns, saline = 2.47 ± 0.24 / session; OT = 2.69 ± 0.24 / session; df = 358, P = 0.146, Wilcoxon rank sum test; M1 number of threats, saline = 1.74 ± 0.39 / session; OT = 2.02 ± 0.40 / session; df = 358, P = 0.371, Wilcoxon rank sum test). **D:** OT treatment increases the cross correlation between M2 staring and M1 turning away (the same as Figure 3D but plotted on a finer time scale). Error bars: mean ± SEM. **E:** OT treatment increases the cross correlation between M1 and M2 staring (the same as Figure 3E but drawn on a finer time scale). Error bars: mean ± SEM. **F:** OT treatment does not alter the cross correlation between M1 staring and M2 turning away (the same as Figure 3F but plotted on a finer time scale). Error bars: mean ± SEM.

Figure S3: Effects of OT and AVP inhalation on behavior. **A:** OT and AVP reduce staring by dominant M1s (low dominance M1, saline = 18.40 ± 2.98 s; OT = 12.78 ± 4.29 s; AVP = 8.93 ± 2.04 s; high dominance M1, saline = 66.56 ± 9.33 s; OT = 40.94 ± 6.32 s; AVP = 35.36 ± 7.91 s; F (2, 24) = 2.16 , P = 0.137, 1-way ANOVA on low dominance M1; F (2, 24) = 4.38 , P = 0.024, 1-way ANOVA on high dominance M1; P = 0.078, multiple comparison, high dominance saline vs OT; P = 0.027, multiple comparison, high

dominance saline vs AVP). Error bars: mean ± SEM; dominance measured by average staring durations under saline. **B:** AVP but not OT reduces staring by dominant M2s (low dominance M2, saline = 18.49 ± 3.06 s; OT = 19.56 ± 2.41 s; AVP = 12.73 ± 2.98 s; high dominance M2, saline = 51.21 ± 7.39 s; OT = 43.11 ± 5.64 s; AVP = 25.72 ± 5.05 s; $F(2, 24) = 1.68$, $P = 0.207$, 1-way ANOVA on low dominance M2; $F(2, 24) = 4.55$, $P = 0.021$, 1-way ANOVA on high dominance M2; $P = 0.622$, multiple comparison, high dominance saline vs OT; $P = 0.018$, multiple comparison, high dominance saline vs AVP). Error bars: mean ± SEM; dominance measured by average staring durations under saline. **C:** OT and AVP increase the cross correlation between M1 and M2 staring (the same as Figure 4D but plotted on a finer time scale). Error bars: mean ± SEM. **D:** The auto correlation of M1 staring does not vary with OT or AVP. Thickness of the curves indicates mean ± SEM.

Figure S4: Effects of OT and AVP injections in ACCg. **A:** For a different population of M1s (C, O, S; n = 60 face-off sessions * 2 treatment conditions), OT injections into ACCg insignificantly reduce staring by M1 (M1 saline = 37.77 ± 3.15 s; OT = 32.47 ± 3.33 s; df = 118, $P = 0.132$, Wilcoxon rank sum test). Black line: gamma fit of saline distribution; magenta line: gamma fit of OT distribution. **B:** For the same population, OT injections into ACCg significantly reduce staring by M2 (M2 saline = 34.70 ± 3.94 s, OT = 25.44 ± 3.66 s; df = 118, $P = 0.038$, Wilcoxon rank sum test). Black line: gamma fit of saline distribution; magenta line: gamma fit of OT distribution. **Insert:** Compared with saline (grey), OT (pink) does not change M1 staring at an empty chair. Error bars: mean ± SEM. **C:** OT and AVP injections into ACCg reduce staring by M1 regardless of dominance order (low dominance M1, saline = 21.99 ± 6.20 s; OT = 7.73 ± 3.18 s; AVP = 6.25 ± 1.92 s; high dominance M1, saline = 73.73 ± 7.49 s; OT = 21.49 ± 3.69 s; AVP = 21.31 ± 3.52 s; $F(2, 15) = 4.34$, $P = 0.033$, 1-way ANOVA on low dominance M1; $P = 0.070$, multiple comparison, low dominance saline vs OT; $P = 0.044$, multiple comparison, low dominance saline vs AVP; $F(2, 15) = 33.31$, $P = 0.000$, 1-way ANOVA on high dominance M1; $P = 0.000$, multiple comparison, high dominance saline vs OT; $P = 0.000$, multiple comparison, high dominance saline vs AVP). Error bars: mean ± SEM; dominance measured by average staring durations under saline. **D:** OT and AVP injections into ACCg reduce staring by dominant but not subordinate M2s (low dominance M2, saline = 22.29 ± 5.43 s; OT = 16.72 ± 4.06 s; AVP = 14.00 ± 3.30 s; high dominance M2, saline = 57.56 ± 5.46 s; OT = 36.59 ± 5.79 s; AVP = 27.94 ± 4.23 s; $F(2, 15) = 0.94$, $P = 0.411$, 1-way ANOVA on low dominance M2; $F(2, 15) = 8.57$, $P = 0.003$, 1-way ANOVA on high dominance M2; $P = 0.031$, multiple comparison, high dominance saline vs OT; $P = 0.003$, multiple comparison, high dominance saline vs AVP). Error bars: mean ± SEM; dominance measured by average staring durations under saline. **E:** OT and AVP injections increase the cross correlation between M1 and M2 staring (the same as Figure 5E but plotted on a finer time scale). Error bars: mean ± SEM. **F:** OT and AVP injections also increase the cross correlation between M2 staring and M1 turning away (the same as Figure 5F but plotted on a finer time scale). Error bars: mean ± SEM.

Supplementary video

A brief video clip (~ 1 minute) taken from one experimental session. M1 (D, left) inhaled OT prior to the experiment, whereas M2 (S, right) did not receive any treatment. A variety of behaviors can be identified from the video clip, including (not necessarily in this order) staring, lip-smacking, looking away, turning away, yawning.

Supplementary Figures S1

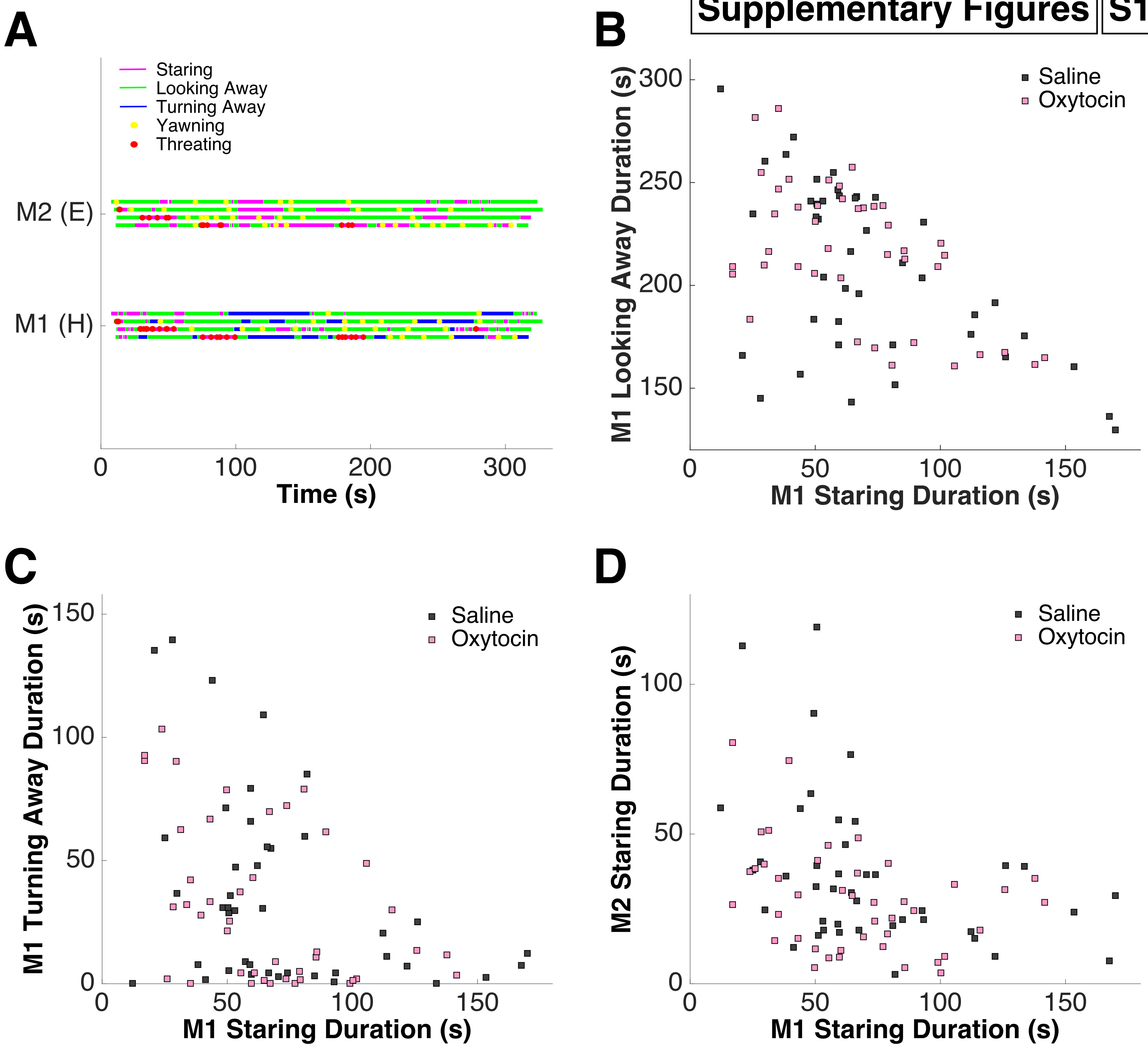

S2

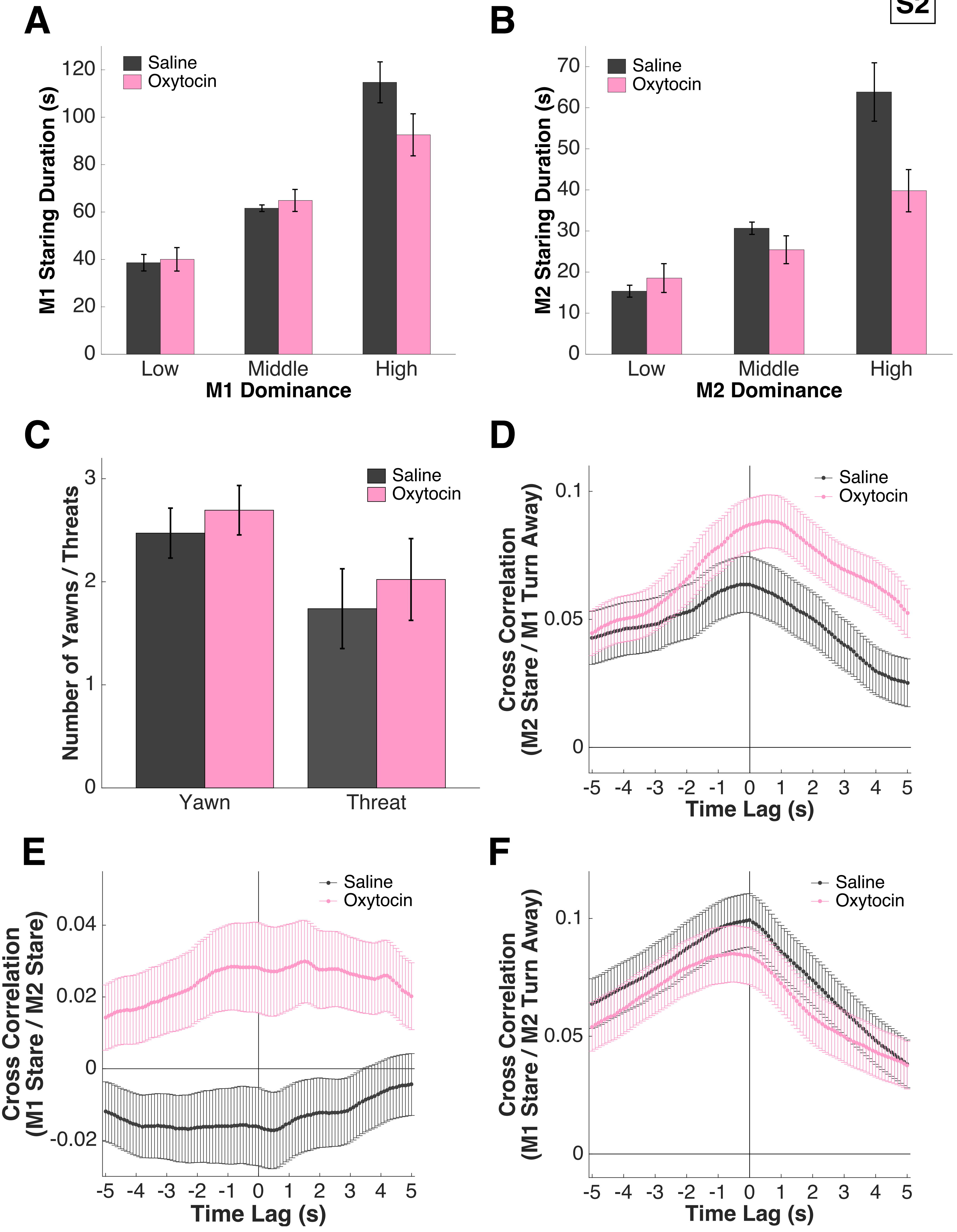

A
Saline
Oxytocin
M1 Staring Duration (s)
0
20
40
60
80
100
120
Low
Middle
High
M1 Dominance
B
Saline
Oxytocin
M2 Staring Duration (s)
0
10
20
30
40
50
60
70
Low
Middle
High
M2 Dominance
C
Saline
Oxytocin
Number of Yawns / Threats
0
1
2
3
Yawn
Threat
D
Saline
Oxytocin
Cross Correlation
(M2 Stare / M1 Turn Away)
0
0.05
0.1
-5 -4 -3 -2 -1 0 1 2 3 4 5
Time Lag (s)
E
Saline
Oxytocin
Cross Correlation
(M1 Stare / M2 Stare)
-0.02
0
0.02
0.04
-5 -4 -3 -2 -1 0 1 2 3 4 5
Time Lag (s)
F
Saline
Oxytocin
Cross Correlation
(M1 Stare / M2 Turn Away)
0
0.05
0.1
-5 -4 -3 -2 -1 0 1 2 3 4 5
Time Lag (s)

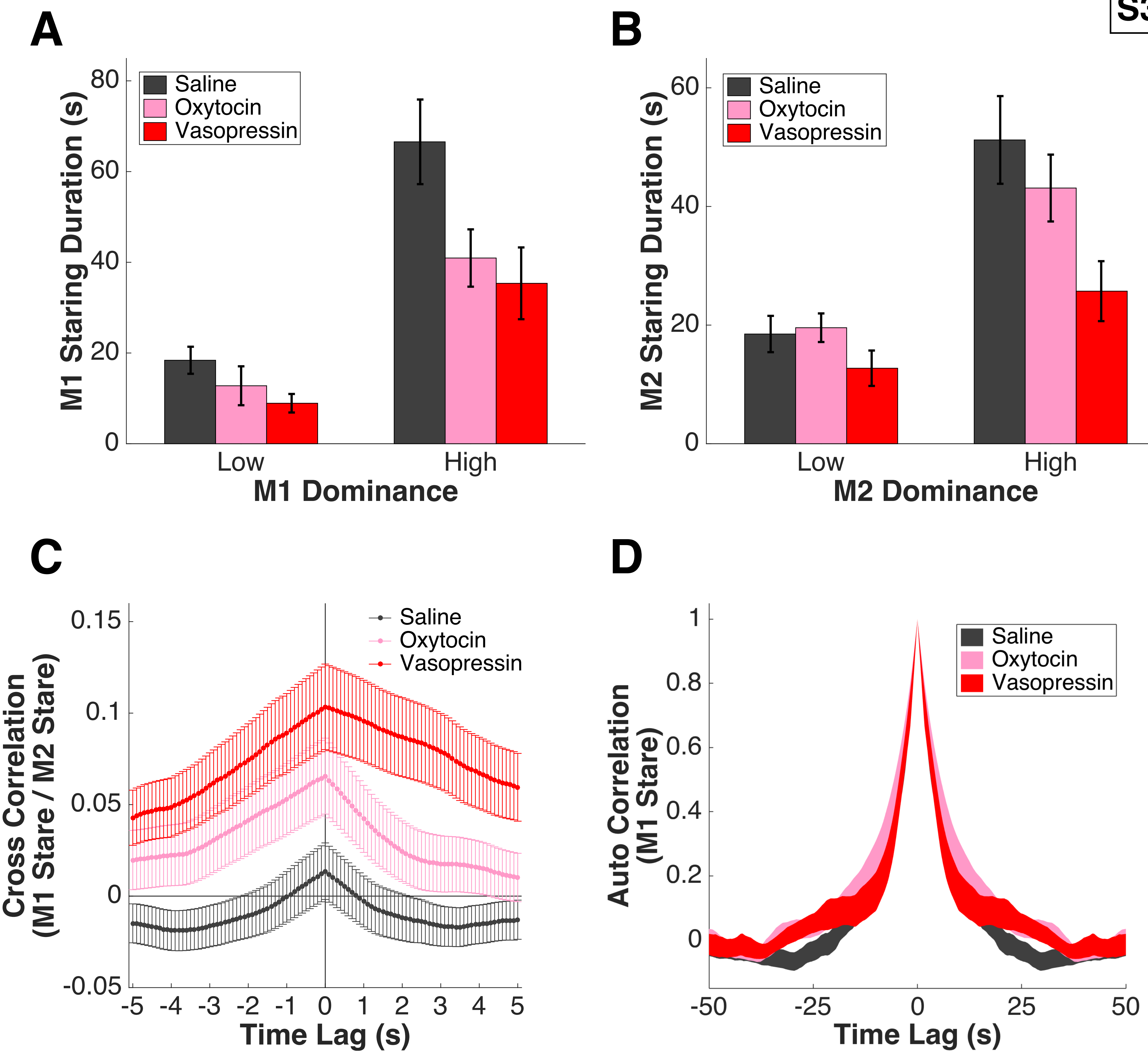
A
Saline
Oxytocin
Vasopressin
M1 Staring Duration (s)
80
60
40
20
0
Low
High
M1 Dominance
B
Saline
Oxytocin
Vasopressin
M2 Staring Duration (s)
60
40
20
0
Low
High
M2 Dominance
C
Saline
Oxytocin
Vasopressin
Cross Correlation
(M1 Stare / M2 Stare)
0.15
0.1
0.05
0
-0.05
-5 -4 -3 -2 -1 0 1 2 3 4 5
Time Lag (s)
D
Saline
Oxytocin
Vasopressin
Auto Correlation
(M1 Stare)
1
0.8
0.6
0.4
0.2
0
-50 -25 0 25 50
Time Lag (s)

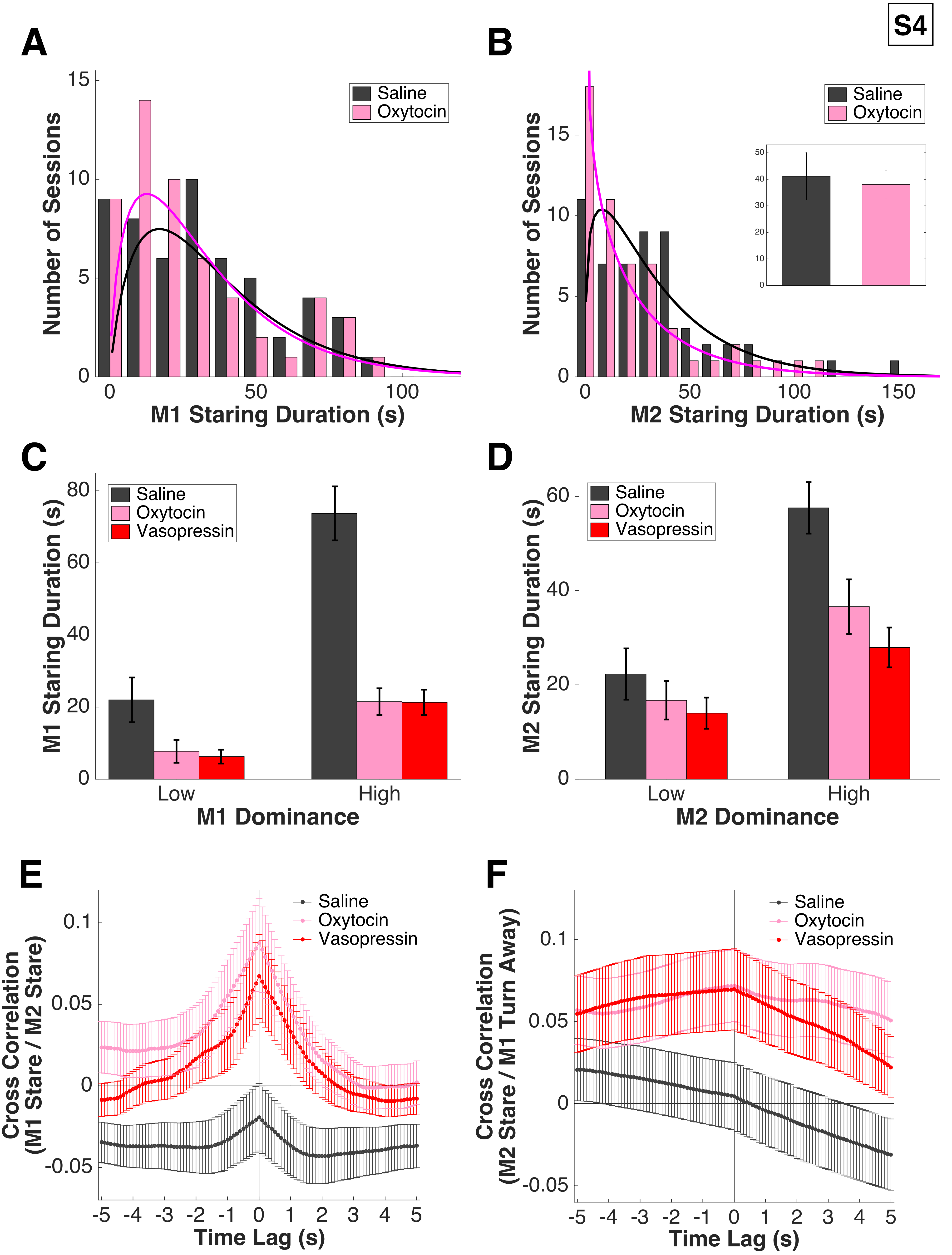
S4
A
Saline
Oxytocin
Number of Sessions
M1 Staring Duration (s)
B
Saline
Oxytocin
Number of Sessions
M2 Staring Duration (s)
C
Saline
Oxytocin
Vasopressin
M1 Staring Duration (s)
Low
High
M1 Dominance
D
Saline
Oxytocin
Vasopressin
M2 Staring Duration (s)
Low
High
M2 Dominance
E
Saline
Oxytocin
Vasopressin
Cross Correlation (M1 Stare / M2 Stare)
Time Lag (s)
F
Saline
Oxytocin
Vasopressin
Cross Correlation (M2 Stare / M1 Turn Away)
Time Lag (s)
